# Are Age Effects in Positivity Influenced by the Valence of Distractors?

**DOI:** 10.1371/journal.pone.0137604

**Published:** 2015-09-14

**Authors:** Maryam Ziaei, William von Hippel, Julie D. Henry, Stefanie I. Becker

**Affiliations:** School of Psychology, University of Queensland, St. Lucia, Brisbane, Australia; University of Leicester, UNITED KINGDOM

## Abstract

An age-related ‘positivity’ effect has been identified, in which older adults show an information-processing bias towards positive emotional items in attention and memory. In the present study, we examined this positivity bias by using a novel paradigm in which emotional and neutral distractors were presented along with emotionally valenced targets. Thirty-five older and 37 younger adults were asked during encoding to attend to emotional targets paired with distractors that were either neutral or opposite in valence to the target. Pupillary responses were recorded during initial encoding as well as a later incidental recognition task. Memory and pupillary responses for negative items were not affected by the valence of distractors, suggesting that positive distractors did not automatically attract older adults’ attention while they were encoding negative targets. Additionally, the pupil dilation to negative items mediated the relation between age and positivity in memory. Overall, memory and pupillary responses provide converging support for a cognitive control account of positivity effects in late adulthood and suggest a link between attentional processes and the memory positivity effect.

## Introduction

A substantial body of evidence shows age-related decline in many cognitive domains, including speed of processing, memory, and attention [1]. Consistent with the possibility that reduced inhibitory control underlies many of these age-related losses [2], age-related declines in control operations such as inhibition have been shown to disrupt memory [3, 4], decision making [5], and various types of social functioning [6–8].

Despite these cognitive losses in late adulthood, emotion regulation tends to remain stable or show small to moderate gains with aging [9, 10]. A central tenet of socioemotional selectivity theory (SST) is that a sense of limited time among older adults leads to increased prioritization of emotion-focused goals over learning or future rewards [11]. It has been argued that this motivational shift may be responsible for the bias toward positive emotional information in attention, memory, and decision-making seen in late adulthood, a phenomenon that has been referred to as the *aging positivity effect*. Whatever the cause of this positivity effect might be (e.g., see aging brain hypothesis [12] or immunosenescence hypothesis [13]), the role of effortful processes in these attentional and memory processes remains unclear.

One possibility is that the aging positivity effect is driven by effortful cognitive control on the part of older adults to regulate their negative emotions [14, 15]. In support of this account, older adults’ executive functioning predicts the magnitude of their positivity effect in both gaze and memory [16]. Additionally, older adults appear to use positivity in gaze as a regulatory mechanism when they are in a negative mood [17], and positivity effects in memory and visual attention disappear when cognitive resources are constrained by dividing attention [18, 19].

Other evidence, however, has not been consistent with the cognitive control account. For example, using pupillometry (pupil size) as an indicator of cognitive effort, it has been found that older adults exert minimal effort to engage in positively biased gaze when experiencing a negative mood [20]. Additionally, Rosler and colleagues [21] found that the positivity effect emerges among older adults with subcortical vascular dementia, a group characterized by prominent losses in cognitive control. Thomas and Hasher [22] also reported that older adults remembered a higher proportion of positive relative to negative words when they were simultaneously performing a numerical discrimination task. Therefore, it is unclear whether the positivity bias among older adults is relatively automatic, imposing only minimal demands on cognitive resources, or whether it might instead be the result of effortful processing.

In addition to the divided-attention tasks mentioned above, another way to identify the underlying cognitive mechanisms of the positivity effect is to measure the distribution of attention towards emotionally valenced items in the presence of distractors. In one study using negative affective priming, less interference from negative than positive distractors was found for older adults relative to their younger counterparts [23]. On the other hand, Ebner and Johnson [24] reported that older adults’ performance was affected by happy emotional distractors more than younger adults. Brassen and colleagues [25] also found that older adults showed increased distractibility to happy faces when more attentional resources were available for processing the distractors. Contrary to these studies, Samanez-Larkin and colleagues [26] found that older adults were more susceptible to interference from a secondary non-emotional task relative to an emotional task. Taken together, the results of these studies indicate that effects of valence on attention may be inconsistent across different methodologies. However, perhaps most importantly in the context of the present study, the primary focus of these studies was on the role of distractors during the early attentional allocation stage, and not how these effects impacted older adults’ subsequent memory. Thus, although there is a large literature focused on the impact of distractors on memory in cognitive aging [27–29], there remains a need to explore how selective attentional processes during encoding influence later memory outcomes for emotional items, and whether effort is implicated in these processes.

### Current study

Using a novel methodological approach, in this study we presented pairs of emotional-emotional or emotional-neutral pictures during encoding and instructed participants to either attend to positive or negative targets in the presence of emotional or neutral distractors. Simultaneous presentation of the distractors with the emotionally valenced targets has the advantage of allowing us to measure whether emotional distractors capture attention, as the extent to which distractors capture attention may vary as a function of age. Therefore, the memory outcome for the targets and the effort expended during encoding of the targets may be differentially impacted by the distractors’ valence.

If positive items automatically attract older adults’ attention, then memory for negative information might be worse in the presence of positive relative to neutral distractors, as positive distractors might capture attention despite intentions to attend to the negative targets. In contrast, if positive information is only more memorable via effortful control, then older adults should be equally capable of ignoring positive and neutral distractors when their task is to focus on negative information. Thus, a cognitive control account of the positivity effect predicts that negative information will be equally well remembered in the presence of positive or neutral distractors.

To further distinguish between these competing accounts, we also measured cognitive effort via pupil dilation during encoding and recognition. Pupil dilation has been shown to function as an indicator of cognitive effort in a number of cognitive and perceptual tasks [31–33]. For instance, increased pupil size in response to enhanced working memory load has been demonstrated in both younger and older adults [34, 35]. Pupil changes were used in the present study to quantify degree of cognitive effort. If the positivity effect relies on effortful control, then older adults should show equivalent pupil dilation when processing negative targets in the presence of positive or neutral distractors. In contrast, the automaticity account predicts that older adults might show larger pupillary responses when processing negative targets in the presence of positive relative to neutral distractors (if positive distractors automatically capture attention and thereby require older adults to exert effortful control over their involuntary tendency to attend to the positive as they attempt to remain focused on the negative targets).

Converging evidence indicates that the positivity effect can emerge from how either positive or negative information is processed (for a review see [36]). That is, people might process the positive information more thoroughly, or alternatively they might engage in effortful suppression processes when attending to negative information. In light of these possibilities, either decreased or increased pupil dilation for the negative items may emerge for older adults, irrespective of the distractors’ valence. Increased pupil dilation for negative items could indicate that older adults are trying to suppress negative items from memory, or alternatively that they are trying to down-regulate their response to the negative items to maintain their positivity focus. Although our paradigm does not allow us to tease apart these two potential mechanisms, by measuring both memory and pupillary responses we can assess whether increased or decreased effort while processing positive or negative information mediates the positivity bias in memory. If enhanced pupillary responses to negative items mediate the memory positivity effect, this would suggest that older adults may be trying to suppress negative items at encoding. On the other hand, if enhanced pupillary responses to positive items mediate the memory positivity effect, this would suggest that older adults may be trying to enhance the encoding of positive items.

## Methods

### Ethics statement

This study was approved by the ethics committee in the School of Psychology at the University of Queensland. All participants were informed of their right to withdraw from the experiment and signed a consent form prior to the experiment. All participants were fully debriefed at the end.

### Participants

Forty one older adults (aged 60–83, *M* = 70.54, *SD* = 4.5) and forty-two younger adults (aged 18–29, *M* = 18.97, *SD* = 2.2) participated in this study. Older adults participated in exchange for $30 AUD and were recruited from the Aging Mind Initiative at the University of Queensland. Younger adults were university students who participated in exchange for course credit. Due to technical issues in acquiring reliable data with an eye tracker, such as glaucoma, multifocal glasses, or excessive blinking during the task, six older adults and five younger adults were excluded. Consequently, 35 older adults (51% male) and 37 younger adults (48% male) contributed to the reported analyses.

All older adults scored 27 or higher on the Mini Mental State Exam [37]. Older adults reported less negative affectivity than younger adults as indexed by the 21-item version of the Depression Anxiety Stress Scales (DASS-21; [38]), *t*(68) = 4.78, *p* < .01, *d* = 1.15. Older adults also reported fewer difficulties regulating their emotions, as indexed by the Difficulties in Emotion Regulation Questionnaire (DERQ; [39]), *t*(62) = 6.23, *p* < .01, *d* = 1.58. Moreover, older adults showed a larger Stroop interference effect than younger adults, *t*(67) = 2.02, *p* < .05, *d* = 0.49, suggesting that they were more readily distracted by the incongruent colour words. None of these differences mediated the positivity effect in memory. Descriptive and inferential statistics for these background measures are presented in Table 1.

**Table 1:** Descriptive and inferential statistics for the background and experimental measures.

Measure	Older adults		Younger adults		Inferential statistics	
	*M*	*SD*	*M*	*SD*	*T*	*df*
Age	70.54	4.53	18.97	2.27		
MMSE	28.50	1.50	-	-	-	-
DERQ	63.91	10.42	84.56	15.83	6.23**	62
DASS-21	4.94	4.27	11.86	7.32	4.78**	68
Interference in Stroop test (%)	27.64	19.80	18.90	15.91	2.02*	67
Memory Positivity Effect	0.12	0.19	0.01	0.18	2.37*	69
FA for negative	0.26	0.19	0.18	0.16	2.01*	69
FA for positive	0.11	0.09	0.13	0.18	0.47	69
Hits for positive	0.77	0.12	0.70	0.20	1.67	69
Hits for negative	0.80	0.14	0.73	0.17	1.78	69

* = *p* < .05,

** = *p* < .01.

MMSE refers to Mini Mental Status Examination;

DERQ refers to Difficulties in Emotion Regulation Questionnaire;

DASS refers to Depression Anxiety Stress Scales.

The percentage of interference in Stroop test refers to ((RTs in incongruent—RTs in neutral trials) / RTs in neutral trials)*100. The overall positivity effect was operationalized as positive accuracy minus negative accuracy.

FA refers to False Alarm rates.

### Materials and procedure

One hundred and ninety images were selected from the International Affective Picture Systems (IAPS, [40]). There were no significant differences between positive and negative pictures in arousal ratings based on Lang et al.’s (2008) database (*t*(69) = 1.68, *p* = .09). The arousal level of the pictures was also used as a covariate in the pupil dilation and memory accuracy analyses reported later in the manuscript. Of these pictures, 70 were negative (valence: *M* = 2.87, *SD* = 0.41; arousal: *M* = 4.90, *SD* = 0.68), 70 were positive (valence: *M* = 7.23, *SD* = 0.45; arousal: *M* = 4.68, *SD* = 0.64), and 50 were neutral (valence: *M* = 4.95, *SD* = 0.26; arousal: *M* = 3.02, *SD* = 0.44). Seventy-eight of these pictures (48 presented during the encoding phase and 30 not previously seen) were used for the recognition task. Equal numbers of pictures were selected from each emotional category for the recognition task (24 positive and 24 negative pictures) and an equal number of items were selected from each experimental condition (12 items of each valence from the conditions with emotional distractors and 12 of each valence from the conditions with neutral distractors). For the not-previously seen pictures, an equal number of items were also selected from each emotional category; positive, negative and neutral (10 items from each category).

Because of differential sensitivity of the pupils to green, red, and blue light [41], a gray scale transformation of pictures was used and presented against a gray background. Pictures were balanced for luminance using a MATLAB program (The source code for this procedure is available online at: https://sites.google.com/site/maryamziaeiwebsite/matlab-programs). The pictures were presented in 512x384 pixels on a 15” Dell computer monitor with 65cm distance from participants’ eyes to the middle of the monitor. Fixation ratio and pupil dilation were recorded using a chin-rest SR EyeLink 1000 eye tracker (SR Research, Ontario, Canada) during the encoding and recognition phases. Pupil size measures were restricted to eye-fixations within the pictures’ coordinates; pupil dilations outside the pictures’ coordinates were excluded from analyses. Prior to the onset of the IAPS pictures, participants performed a 9—point eye calibration to ensure that the eye-tracker was recording the position of the eyes accurately from different points on the screen. After calibration, the 9—point validation procedure was applied to ensure that the calibration procedure had been performed correctly. During calibration and validation, participants were asked to focus on each fixation point until it disappeared and then move their eyes to the next fixation point on the screen. This procedure was then followed by presentation of a black and then a white screen for 10 seconds each to provide baseline measures of pupil dilation prior to the encoding phase. Participants’ pupil responses were recorded continuously while the pictures were presented. However, only pupillary responses and fixation ratio during the picture presentations were included in the analyses. Eye tracker data recorded outside of the pictures and during the fixation crosses presented between each picture were discarded. Drift correction procedures were applied prior to each trial to ensure that participants’ eyes were focused on the center of the screen prior to being shown the experimental stimuli.

### Experimental design

#### Encoding task

**Fig 1:**
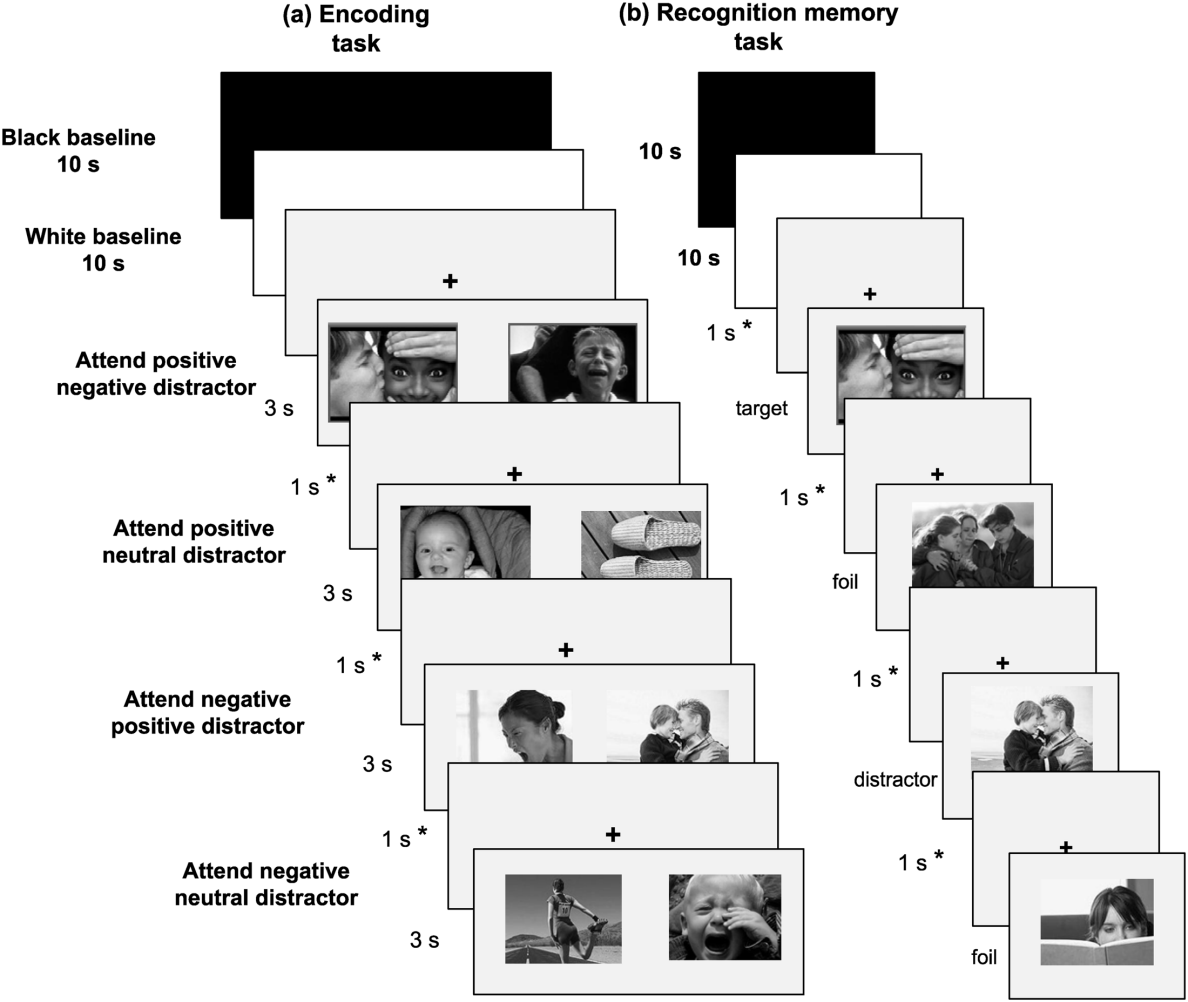
Example of experimental design. Each screen consisted of a pair of pictures presented side by side for 3 seconds during encoding (Panel a). During recognition (Panel b), participants were asked to indicate whether they had seen the picture before. Due to the drift correction applied after each trial, the maximum length of the fixation crosshair varied between subjects. * indicates the minimum length of 1 second for each fixation crosshair presentation.

The encoding tasks used in conjunction with the eye-tracker consisted of four within-subjects conditions. The order of conditions was counterbalanced across participants. Pictures were presented in pairs of emotional-emotional or emotional-neutral stimuli with the target photo randomly placed on either side of the screen. Participants were instructed to attend to the target (positive or negative) and ignore the distractors (emotional or neutral). They were instructed to attend to positive images for 40 trials and to negative images in another 40 trials. Within each of these conditions, 20 of the distractors were neutral in valence and 20 were of the opposite valence as the target image. Participants received an instruction prior to each condition that was followed by presentation of the pictures. Each pair of pictures was presented for 3 seconds with a minimum of 1 second crosshair between presentations to realign participants’ gaze to the center of the screen (Fig 1a).

#### Recognition memory task

After completion of the encoding and following a ten-minute filler task, participants performed an incidental recognition memory task. They were asked to indicate whether each picture was previously shown during the encoding phase by pressing one of two keys on the keyboard at their own pace (Fig 1b). During the recognition memory phase, pupil sizes were recorded. Therefore, the calibration procedure was applied again before the recognition task to control for possible memory-load effect on pupil dilations, with baseline measurement of pupil dilations during 10-second presentations of black and white screens. The pupillary responses to these baseline measures were used for the transformation procedure applied to the recognition memory data (see below). Experimental sessions ranged from 90 minutes to 2 hours inclusive of a 15-minute break. Due to the drift correction applied after each trial, the maximum duration of the crosshair varied between subjects during both encoding and recognition tasks.

### Statistical analyses

#### Memory discrimination

For the recognition data, signal detection analyses were used to calculate accuracy scores by subtracting false alarms (FAs) from hits (as in [42]). Due to the presence of extreme values in hits and FA scores, a loglinear approach was used as proposed by Stanislaw and Todorov [43]. This approach involved adding 0.5 to the number of hits and FAs and adding 1 to the number of signal and noise trials before calculating the hit and FA rates. Thus, memory accuracy scores were computed as loglinear hits minus loglinear false alarms. The false alarms were subtracted from the hits based on the emotional category of the targets’ valence, irrespective of the distractors’ valence.

#### Pupillary responses

To control for individual differences between and among younger and older adults in pupil dilation at baseline, we applied transformation procedures similar to those used by Allard et al. [20]. Specifically, pupil diameters to the white screen were subtracted from pupil diameters to each picture, and this difference score was divided by the difference between the pupil diameters to the black and the white screen ([current pupil diameter-minimum overall pupil diameter] / [maximum overall pupil diameter—minimum overall pupil diameter]. Here, higher scores indicate a greater range of change in pupil dilation (larger pupil diameter, thus increased cognitive effort) while viewing a particular image. This transformation procedure was applied for both encoding and recognition memory phases.

#### Fixation ratio

Although participants were instructed to attend to the targets and ignore the distractors, we also calculated the ratio of gaze fixations to targets and distractors, with fixations defined as gaze orientations to a particular point on the screen for at least 100 ms [44]. This ratio reflects the amount of time participants spent viewing the targets relative to the distractors in each condition. The fixation ratio was calculated using the procedure described by Isaacowitz et al. [45]: (Target—distractors)/ (Targets + distractors).

#### Positivity score

Positivity scores in the presence of emotional and neutral distractors were computed separately by calculating the difference in memory between positive and negative targets in these two conditions. Overall positivity scores were calculated by averaging these two conditions (Table 1). Recognition memory and pupillary responses were subjected to mixed-model analyses of variance (ANOVAs) with age group as between-subjects factors and target valence and distractor types as within-subjects factors.

## Results

### Gaze fixation times for targets relative to distractors

**Fig 2:**
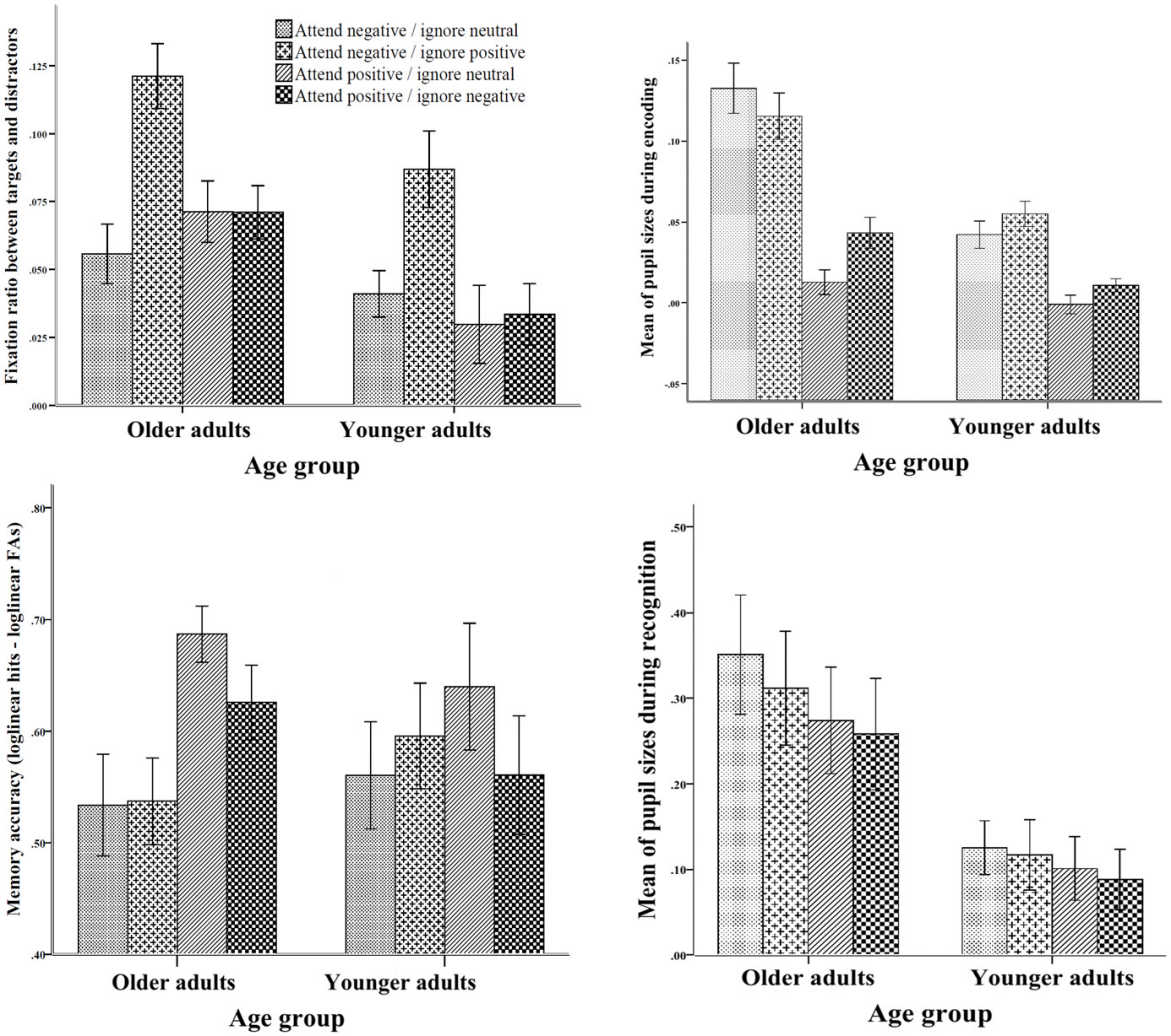
Eye-tracker and memory findings during encoding and recognition phases. Panels a and b represent fixation ratios and pupillary responses for targets, respectively. Panel c represents the memory accuracy for targets from each condition during recognition, and Panel d represents pupillary responses for targets during recognition. Bars represent one standard error of the mean (SEM).

A 2 (distractor type) x 2 (target valence) x 2 (age group) ANOVA on fixation ratios revealed a main effect of target valence, *F*(1,67) = 10.22, *p* < .05, η_p_^2^ = .88, and a main effect of distractor type, *F*(1,67) = 14.91, *p* < .01, η_p_^2^ = .96, indicating greater fixation times on negative items than positive targets and greater fixation times on emotional distractors than neutral distractors (Fig 2a). The interaction between target valence and distractor type was also significant, *F*(1,67) = 12.74, *p* < .01, η_p_^2^ = .94. Simple effects analysis revealed that fixation ratios were larger when negative items were presented with positive distractors than with neutral distractors, *t*(68) = 5.52, *p* < .01, *d* = 1.33. No significant differences in fixation ratios emerged for positive targets as a function of whether they were presented with negative or neutral distractors, *t*(69) = 0.38, *p* = .70, *d* = 0.09. These results suggest that both age groups fixated more on the negative targets when they were presented with positive distractors but the fixation ratio for positive targets was not influenced by the valence of the distractors. A main effect of age group also emerged, *F*(1,67) = 9.84, *p* < .01, η_p_^2^ = .87, with older adults spending more time fixated on the pictures rather than somewhere else compared to younger adults, independent of the valence of the pictures. These data suggest that older adults are putting greater effort into encoding than their younger counterparts by fixating more on the relevant targets. To test this possibility, we reran the pupil dilation analysis using total fixation ratio as a covariate. The results showed that the main effect of age was no longer significant

Additional analyses, including the number of switches between the targets and distractors as well as the latency of the initial fixation, are presented in S1 Table.

### Pupillary responses for targets during encoding

The next stage in the analyses was to assess whether there were differential effects of age on pupil dilation (reflecting the level of cognitive effort) across the different valence and distractor conditions. With regard to pupillary responses during encoding, a 2 (age group) x 2 (target valence) x 2 (distractor type) repeated measures ANOVA on pupil size revealed a main effect of target valence, *F*(1,66) = 99.95, *p* < .01, η_p_^2^ = 1.0, with larger pupil sizes for negative than positive targets (Fig 2b). The main effect of age group was also significant, *F*(1,66) = 32.14, *p* < .01, η_p_^2^ = 1.0, with older adults showing larger pupil dilations compared to younger adults (Fig 2b). A significant interaction also emerged between target valence and age group, *F*(1,66) = 14.08, *p* < .01, η_p_^2^ = .95, such that although pupil sizes were larger for negative items than positive items in both groups, the magnitude of this effect was larger in the older group (*M*_*negative*_ = .13, *SD* = .08 vs. *M*_*positive*_ = .02, *SD* = .04), *t* (31) = 7.27, *p* < .01, *d* = 1.74, than the younger group (*M*_*negative*_ = .05, *SD* = .04 vs. *M*_*positive*_ = .005, *SD* = .02), *t* (35) = 7.28, *p* < .01, *d* = 1.42.

The interaction between target valence and distractors was significant, *F*(1,66) = 5.30, *p* = .02, η_p_^2^ = .62, with larger pupil sizes for positive targets presented with negative distractors relative to neutral distractors, *t*(69) = 2.63, *p* < .01, *d* = 0.42, but no significant difference in pupillary responses to the negative targets presented with neutral or positive distractors, *t*(69) = .01, *p* = .99, *d* < 0.01. The three way interaction was also significant, *F*(1,66) = 5.77, *p* = .02, η_p_^2^ = .65, such that older (but not younger) adults showed a significant difference in pupil dilation to positive targets presented with neutral and negative distractors (Fig 2). None of the other effects were significant, all *F*s < 1. The lack of an effect of positive distractor valence on pupil size suggests that positive distractor images were not automatically attracting older adults’ attention. The increased pupil size shown by older relative to younger adults when encoding negative target images suggests a role for effortful processing either in inhibiting the negative items from memory and/or in the regulatory mechanisms recruited during encoding.

### Recognition memory for targets

A 2 (age group) x 2 (target valence) x 2 (distractor type) ANOVA on recognition accuracy scores revealed a main effect of target valence, *F*(1,68) = 10.16, *p* <.01, η_p_^2^ = .88, with better memory for positive items compare to negative items. This main effect was qualified by the predicted interaction between target valence and age group, *F*(1,68) = 4.81, *p* = .03, η_p_^2^ = .06 (see Fig 2c). Simple effects analyses revealed greater memory accuracy for positive targets than negative targets among older adults (*M*_*positive*_ = .65, *SD* = .15 vs. *M*_*negative*_ = .53, *SD* = .23), *t*(34) = 3.67, *p* < .01, *d* = 0.61, but not among younger adults (*M*_*positive*_ = .57, *SD* = .35 vs. *M*_*negative*_ = .55, *SD* = .30), *t*(35) = 0.69, *p* = .49, *d* = 0.06.

A significant interaction also emerged between target valence and distractor types, *F*(1,68) = 8.49, *p* < .01, η_p_^2^ = .11. Simple effects analyses revealed no differences in memory accuracy of negative targets when presented with positive vs. neutral distractors, *t*(69) = 0.86, *p* = .38, *d* = 0.03, but greater memory accuracy for positive targets when presented with neutral vs. negative distractors, *t*(70) = 3.66, *p* < .01, *d* = 0.87. No main effects emerged for distractor type, *F*(1,68) = 2.93, *p* = .09, η_p_^2^ = .04, or age group, *F*(1,68) = 0.01, *p* = .90, η_p_^2^ = .05, nor was there any interaction between distractor type and age group, *F*(1,68) = 0.05, *p* = .81, η_p_^2^ = .05, nor was there a three-way interaction, *F*(1,68) = 0.65, *p* = .42, η_p_^2^ = .12. Because positive distractors did not interfere with recognition memory of the negative targets more than neutral distractors, these results suggest that older adults do not differ from younger adults in this regard, and their attention was not automatically attracted to the positive images.

In addition to the analyses focused on memory accuracy, we also analyzed the hits and FA rates separately. The results from a 2 (age group) by 2 (positive vs. negative FAs) mixed model ANOVA showed a main effect of FA valence, *F* (1,69) = 34.00, *p* < .01, η_p_^2^ = .33, with higher FA rates for negative relative to positive items. The interaction between age group and FA valence was also significant, *F* (1,69) = 9.28, *p* < .01, η_p_^2^ = .11, such that older adults showed more FAs for negative items relative to younger adults, *F* (1,69) = 4.04, *p* < .05, η_p_^2^ = .05, but did not differ from younger adults in positive FAs, *F* (1,69) = 0.22, *p* = .63, η_p_^2^ = .003 (see Table 1). Similar analyses were performed for hit rates and none of the effects reached significance, all *F*s < 1.

### Covariate analyses with arousal ratings

Given the potential sensitivity of pupil dilation to the arousal level of the pictures, the pupillary analysis was also conducted with the arousal level of the pictures as a covariate. The age by target valence interaction remained significant, *F*(1,63) = 6.03, *p* <.05, η_p_^2^ = .12, suggesting that the interaction was not a function of different arousal levels of the positive and negative pictures.

A similar analysis was also conducted on memory accuracy scores using the arousal level of the pictures as a covariate. The results continued to reveal a significant age by target valence interaction, *F*(1,63) = 6.87, *p* <.05, η_p_^2^ = .13, suggesting that the memory positivity effect that emerged among older adults relative to younger adults was also not a function of differential arousal levels of the pictures.

### Mediation analysis

**Fig 3:**
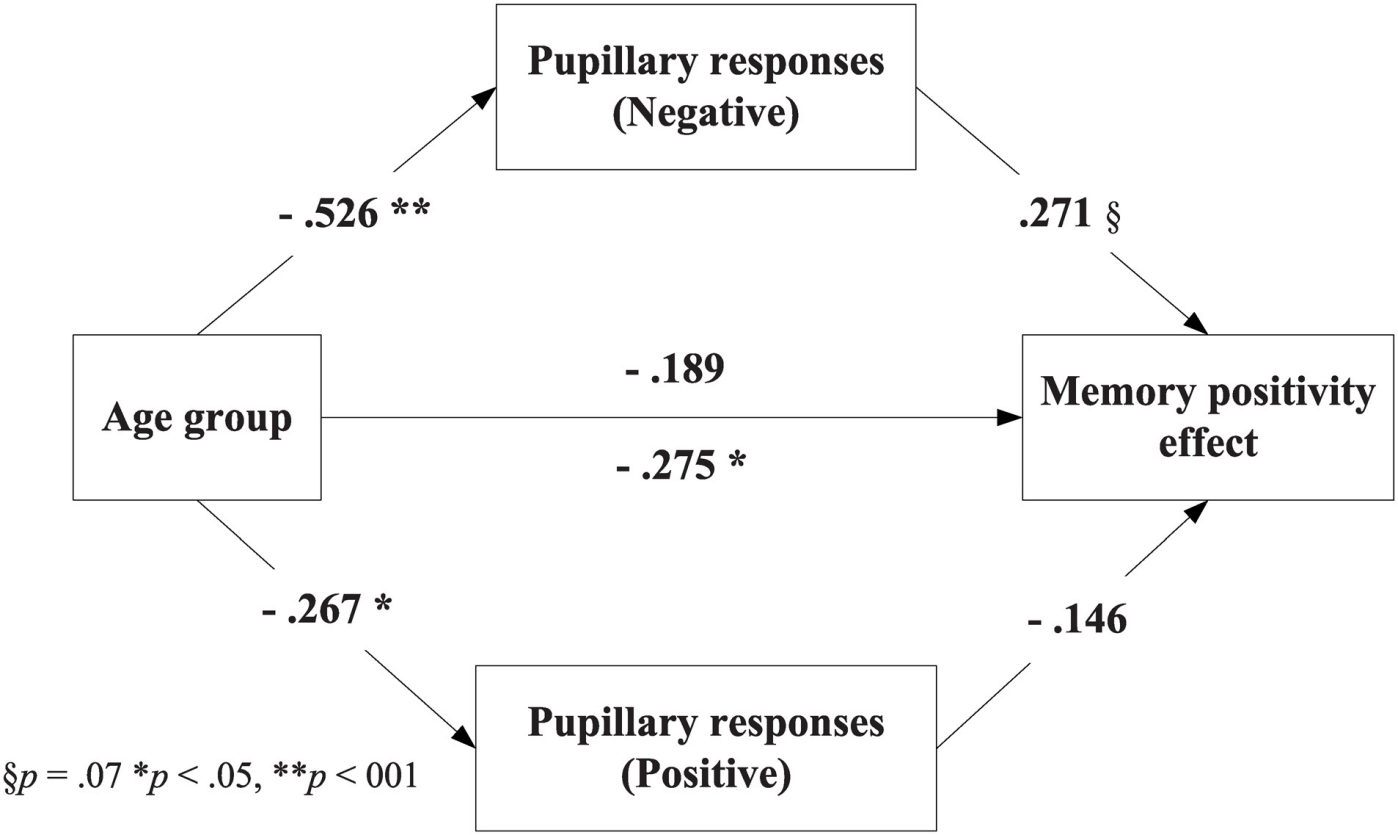
Mediation of the effect of age on the memory positivity effect via pupillary responses. The numbers along the paths represent standardized regression coefficients. The coefficient below the path from age group to memory positivity represents the direct effect with no mediator in the model; the coefficient above the path represents the effect when pupillary dilation was included as a mediator.

Next, we examined whether the relation between age group and the memory positivity effect is mediated by the pupillary responses obtained from the encoding phase. The results indicated that the relationship between age group and positivity effect in memory was mediated by the cognitive effort exerted during encoding of negative items, but not positive items. The standardized indirect effect was measured using bootstrapping procedure with 10,000 resamples, and found to be-.05, 95% CI [-.13, -.006] (see Fig 3). This result suggests that the memory positivity effect was mediated by the amount of the effort exerted during encoding of negative items, possibly in an effort to suppress negative information from memory.

### Pupillary responses for targets during recognition

Next, we analyzed the pupillary responses during recognition for the target items as a function of the distractor types with which they were presented. A 2 (age group) x 2 (target valence) x 2 (distractor type) ANOVA on pupil sizes during the recognition task revealed a main effect of target valence, *F*(1,66) = 23.69, *p* < .01, η_p_^2^ = .99, with larger pupil sizes for negative than positive targets. A significant interaction between target valence and age group also emerged, *F*(1,66) = 4.28, *p* = .04, η_p_^2^ = .53. Simple effects analyses revealed larger pupil dilations in response to negative than positive targets, and this effect was larger among older adults (*M*_*negative*_ = .33, *SD* = .38 vs. *M*_*positive*_ = .26, *SD* = .36), *t*(32) = 4.55, *p* < .01, *d* = 0.18, than younger adults (*M*_*negative*_ = .12, *SD* = .20 vs. *M*_*positive*_ = .09, *SD* = .20), *t*(33) = 2.17, *p* = .03, *d* = 0.15 (Fig 2d). Given that the pattern of pupillary responses during recognition resembled the pattern of results from encoding, it seems that greater cognitive effort was required during encoding and recognition of negative items relative to positive items for older adults.

## Discussion

The present study investigated the effect of instructed attention towards emotional targets in the presence of emotional versus neutral distractors during encoding, and examined the impact of these distractors on the subsequent recognition memory performances of older and younger adults. Consistent with prior research, older adults showed a memory positivity effect such that they were more accurate in their recognition of positive than negative items, whereas younger adults did not show this effect. The present study also extends the prior literature (for a review see [30]) by showing that this memory positivity effect was independent of the valence of distractors during encoding. In particular, memory for negative information was not influenced by whether the distractors were positive or neutral, which suggests that positive items do not automatically capture the attention of older adults when they are intentionally directing attention to negative items. The results also provide evidence that the cognitive effort older adults exert while encoding negative items mediates the relation between age and the memory positivity effect.

Previous studies have tested the role of cognitive control in the processing of emotionally valenced items using a range of experimental designs. Here we used a novel approach to examine the link between attention and memory and to assess whether distractors that vary in emotional valence differentially impact attention and memory for targets. The amount of effort required for processing positive distractors in particular, allowed us to infer whether cognitive control or automaticity accounts most readily explain positivity effects in memory. By presenting emotional-emotional and emotional-neutral pairs of stimuli during encoding, we were able to compare the effect of emotional versus neutral distractors on two dependent measures (i) participants’ memory of the targets, and (ii) their pupillary responses. The lack of a significant difference in memory for negative targets when presented with positive or neutral distractors suggests that the presence of positive distractors did not automatically interfere with participants’ encoding of the negative targets. These results therefore suggest that cognitive control plays a role in the aging positivity effect in memory. Additionally, negative distractors had a more detrimental effect than neutral distractors on memory for positive targets across both age groups. Taken together, these results are consistent with previous findings showing the resource-dependency of the positivity effect in participants who recruit brain regions known to be particularly important for cognitive control (e.g. frontal regions [25]).

Consistent with the memory data, older adults did not show differential pupillary responses as a function of distractor valence when viewing negative pictures. These results indicate that positive distractors did not automatically attract older adults’ attention away from negative targets, and thus did not require older adults to inhibit this automatic response in an effort to stay focused on the target items. Although the usefulness of pupillary responses to detect subtle changes in cognitive effort apparently decreases with age [34], pupillary responses in the current experiment were sensitive to target valence among older adults. Thus, these data are consistent with the recognition memory data in supporting a cognitive control account of the memory positivity effect.

It remains unclear, however, exactly why older adults showed a larger pupillary response to negative relative to positive targets. There are at least two possible interpretations of this pattern of findings. First, the processing of negative items might have required greater cognitive effort to down regulate negative emotions by older adults. Second, greater effort during the encoding of negative items may have reflected older adults’ efforts to suppress their memory for the negative images, resulting in better memory for the positive images during the recognition task. The increased FA rates and the mediation results support this latter interpretation.

We should note, however, that although the results remained unchanged when the arousal ratings of the pictures were used as a covariate in the analyses, it is still possible that pupillary responses for negative items might be related to differences in arousal levels. According to the negativity bias principle, “negative events are more salient, potent, and dominant than positive events [46]” and therefore, a stronger reaction may be elicited by negative items relative to positive items [47]. Thus, the impact of arousal on pupil dilation and memory would benefit from further investigation, although it seems unlikely that the results of the current research reflect differences in arousal between negative and positive items.

Alternatively, the larger pupillary responses during encoding of negative items by older adults might reflect emotion-regulatory mechanisms. Such a possibility would be in line with previous studies suggesting that pupillary responses reflect regulation during negative moods [17, 20]. As regulatory mechanisms often demand cognitive effort, it is possible that the pupillary responses reflected the cognitive effort exerted for down-regulating negative emotions. This interpretation is consistent with three other research findings. First, previous studies in the emotion regulation literature indicate that emotion regulation imposes substantial demands on cognitive control operations [48, 49]. Second, older adults appear to be well equipped to regulate their emotions in the earliest stages of the emotion-generative process [10, 50, 51]. Third, a number of functional neuroimaging studies suggest that older adults use their cognitive control regions—prefrontal cortex regions—when encoding or processing negative items [52], which could reflect an effort to down-regulate negative emotions.

With regard to the fixation ratios, greater fixation time was spent on the negative targets presented with positive rather than neutral distractors across both age groups. Similar to prior research [53], older adults were able to attend to the relevant items and ignore the irrelevant ones when instructed to do so, despite the fact that this ability tends to be impaired in late adulthood [53]. The converging results from fixation ratio, pupil dilations, and memory suggest that older adults were able to follow the instruction to attend to the targets. Although previous studies suggest that the positivity effect emerges when no experimental constraints are applied [36], our current findings suggest that the type of instruction might have an impact on the positivity effect. Goal-directed instructions to process the relevant targets in this study might have supported the involvement of top-down processes in the positivity effect [36]. It remains for future studies to investigate how different types of regulatory mechanisms during encoding are linked to the positivity bias in memory.

One limitation of this study was that we did not include neutral targets. Future studies are therefore needed that investigate memory for neutral targets in the presence of emotional distractors. Moreover, further investigation using participants’ own subjective ratings of arousal and valence is needed. Evidence indicates that there are some differences between older and younger participants in arousal rating of IAPS pictures [54]. Therefore, future studies should investigate whether participants’ own subjective ratings of arousal for emotionally valenced items influence pupillary responses. Different methodological tools such as EEG or fMRI will also be important for capturing the underlying neural mechanisms of the aging positivity effect (for example see [25]).

In conclusion, three primary findings emerged: 1) Older adults’ recognition memory for negative items was not affected by the valence of distractors during encoding; suggesting that greater attention to positive information among older adults is not a result of automatic attention capture. 2) Consistent with these memory findings, no pupillary changes emerged as a function of distractor valence at either encoding or recognition of negative targets, again suggesting that positive distractors did not automatically capture attention from its intended target. 3) Pupillary responses to negative items mediated the relationship between age and the memory positivity effect, suggesting that older adults may have suppressed the negative items from their memory during encoding. Taken together, these data provide further insights into the link between attentional processes and later recognition memory, and provide support for a cognitive control account during encoding that leads to a memory positivity effect in late adulthood.

## Supporting Information

S1 Table Descriptive and inferential statistics for the additional analyses of the eye-tracker data. The number of gaze switches between targets and distractors as well as initial fixation durations on the targets for each condition are reported in this table.
(DOCX)
