# Supplementary material for: Are Age Effects in Positivity Influenced by the Valence of Distractors?
Source: PLoS One. 2015 Sep 14;10(9):e0137604. doi: 10.1371/journal.pone.0137604 (PMC4569566; doi:10.1371/journal.pone.0137604)
Supplement: S1 Table — The number of gaze switches between targets and distractors as well as initial fixation durations on the targets for each condition are reported in this table. (DOCX) [file pone.0137604.s001.docx]

**S1 Table. Descriptive and inferential statistics for the additional analyses on the eye-tracker data**

| Measure | Older adults | | Younger adults | | Inferential statistics | |
| --- | --- | --- | --- | --- | --- | --- |
|  | *M* | *SD* | *M* | *SD* | *t* | *df* |
| ***Gaze switches*** | | | | | | |
| Attend negative/ignore neutral | 1.80 | 0.34 | 1.84 | 0.32 | 0.51 | 70 |
| Attend negative/ignore positive | 1.68 | 0.23 | 1.78 | 0.33 | 1.49 | 70 |
| Attend positive/ignore neutral | 2.07 | 0.32 | 2.06 | 0.32 | 0.13 | 70 |
| Attend positive/ignore negative | 2.10 | 0.44 | 2.02 | 0.37 | 0.90 | 70 |
| ***Initial fixation duration in ms. (latency)*** | | | | | | |
| Attend negative/ignore neutral | 1081.80 | 364.58 | 1661.57 | 479.16 | 5.75** | 70 |
| Attend negative/ignore positive | 1060.39 | 357.36 | 1689.64 | 509.54 | 6.03** | 70 |
| Attend positive/ignore neutral | 1055.60 | 355.85 | 1634.87 | 478.76 | 5.80** | 70 |
| Attend positive/ignore negative | 1040.10 | 358.65 | 1663.04 | 471.87 | 6.28** | 70 |

**= *p* < .005
